# Interfering Expression of Chimeric Transcript* SEPT7P2-PSPH* Promotes Cell Proliferation in Patients with Nasopharyngeal Carcinoma

**DOI:** 10.1155/2019/1654724

**Published:** 2019-04-01

**Authors:** Jing Wang, Guo-Feng Xie, Yuan He, Ling Deng, Ya-Kang Long, Xin-Hua Yang, Jiang-Jun Ma, Rui Gong, Wen-Jian Cen, Zu-Lu Ye, Yi-Xin Zeng, Hai-Yun Wang, Jian-Yong Shao

**Affiliations:** ^1^State Key Laboratory of Oncology in South China, Collaborative Innovation Center for Cancer Medicine, Guangdong Key Laboratory of Nasopharyngeal Carcinoma Diagnosis and Therapy, Sun Yat-sen University Cancer Center, No. 651 Dongfeng East Road, Guangzhou 510060, China; ^2^Department of Molecular Diagnostics, Sun Yat-sen University Cancer Center, Guangzhou, 510060, China

## Abstract

**Introduction:**

Nasopharyngeal carcinoma (NPC) is a distinct type of head and neck cancer which is mostly prevalent in southern China. The development of NPC involves accumulation of multiple genetic changes. Chromosomal translocation is always thought to be accompanied with the fusion chimeric products. To data, the role of the fusion chimeric transcript remains obscure.

**Materials and Methods:**

We performed RNA sequencing to detect the fusion genes in ten NPC tissues. Sanger sequencing and quantitative RT-PCR were used to measure the level of the fusion chimeric transcript in NPC tissues and cell lines. The functional experiments such as CCK8 assay, colony formation, and migration/invasion were conducted to analyze the role of this transcript in NPC* in vitro*.

**Results:**

We demonstrated that the chimeric transcript* SEPT7P2-PSPH* was formed by trans-splicing of adjacent genes in the absence of chromosomal rearrangement and observed in both NPC patients and cell lines in parallel. Low-expression of the* SEPT7P2-PSPH* chimeric transcript induced the protein expression of* PSPH* and promoted cell proliferation, metastasis/invasion, and transforming ability* in vitro*.

**Conclusions:**

Our findings indicate that the chimeric transcript* SEPT7P2-PSPH* is a product of trans-splicing of two adjacent genes and might be a tumor suppressor gene, potentially having the role of anticancer activity.

## 1. Introduction

Nasopharyngeal carcinoma (NPC) presents regional differences in incidence, such as high incidences in North Africa and Southern China [1]. More than 60% of NPC patients at diagnosis are in late stages or even present with metastasis although many biomarkers were reported to be useful for early detection such as Epstein-Barr virus (EBV) antibodies [2]. Therefore, there is still an urgent need to identify the biomarkers for cancer diagnosis and therapeutic guidance.

Gene fusions derived generally from chromosomal rearrangements are considered as cancer-related genetic events, and their products including RNAs and proteins are identified as specific diagnostic and therapeutic targets for cancers such as hematologic malignancy and lung adenocarcinoma [3, 4]. However, certain chimeric transcripts can be formed without chromosomal rearrangements. Previous studies demonstrated that a fusion transcript named* SLC45A3-ELK4* was formed with exon 1 of* SLC45A3* and the last 4 exons of* ELK4 *[5, 6], which acted as oncogenes in prostate tumor cells without chromosomal changes [7]. Additionally, many studies showed a plenty of fusion transcripts present in normal and/or tumor cells [8–12]. Therefore, fusion genes might be occurred in normal or tumors cells with/without chromosomal changes. Although previous studies have already reported some tumor specific fusion genes in NPC, all of them occur accompanied with the rearrangement of chromosomes [13, 14].

Herein, we carried out RNA sequencing and discovered a novel type of fusion gene, trans-splicing chimeric transcript* SEPT7P2-PSPH*. In addition, we performed* in vitro* studies to demonstrate that the ability of cell invasion, migration, proliferation, colony formation, and transforming were activated when the expression of the transcript was decreased, indicating that* SEPT7P2-PSPH* act as the tumor suppressor gene role in NPC tumorigenesis.

## 2. Materials and Methods

### 2.1. Primary Tumors and Pair-End RNA Sequencing

Ten formalin fixed paraffin embedded (FFPE) NPC samples were retrieved from the Department of Pathology of Sun Yat-sen University Cancer Center (SYSUCC, Guangzhou, China). All tumor samples were reviewed by two experienced pathologists. Total RNA were extracted from ten FFPE tissues with RNeasy FFPE Kit (QIANGEN, Hilden, Germany) according to the manufacturer's instructions for RNA sequencing. cDNA libraries were prepared according to the standard protocol provided by the mRNA-seq sample Pre Kit (Illumina Inc, California, USA) and sequenced (90 nt paired-end) on the Illumina Hi-seq 2000. To identify fusion genes from paired-end RNA sequencing, the data were analyzed by the computational pipeline called SOAPfusion, which uses clusters of discordant paired-end alignments to inform a split-read alignment analysis for finding fusion boundaries [15]. The UCSC* H. sapiens* reference genome (build hg19) was used for alignments. The written informed consent was obtained from each patient and this study was approved by Ethics Committee and Institutional Review Board of SYSUCC.

### 2.2. Cell Cultures, Reverse Transcription and Sanger Sequencing

CNE1 and 6-10B used in the study were cultivated in RPMI 1640 (Invitrogen, Carlsbad, CA, USA) supplemented with 10% FBS (Gibco, California, USA) in a humidified 5% CO_2_ incubator at 37°C. All cultivations involved have been sustained within 6 months. Total RNA was extracted using Trizol (Invitrogen, Carlsbad, CA, USA) according to the manufacturer's instructions. Reverse transcription was subsequently performed with PrimeScript RT reagent Kit with gDNA Eraser (TaKaRa, Kusatsu, Shlga, Japan). The products were employed with specific primers for Sanger sequencing to confirm potential fusion transcripts. The primers were shown as follows:

 
*SEPT7P2-PSPH* 1-sense: 5′-TTACGACTTCTCGGTCTTCGG-3′
 
*SEPT7P2-PSPH* 1-anti-sense: 5′-CGTCCTCAACGCCACAGATT-3′

### 2.3. Quantitative RT-PCR

Quantitative RT-PCR was performed using the ABI 7500 Real-time PCR system (Applied Biosystems, California, U.S.A) and SYBR Premix EX Taq (TaKaRa, Kusatsu, Shlga, Japan). All reactions were carried out in triplicate. The housekeeping gene* GAPDH* was used as normalization control. The relative expression levels of target gene* SEPT7P2-PSPH* were calculated by 2^−△△*Ct*^ method, in which △*Ct* = mean* Ct SEPT7P2-PSPH *– mean* Ct* control, where* Ct* values are the cycle threshold for each sample [16]. Specific primers were shown as follows:

 
*SEPT7P2-PSPH* 2-sense: 5′-ACCTGAGCCTGGGAGGAAA-3′
 
*SEPT7P2-PSPH* 2-anti-sense: 5′-GTCAACATCAAAACACACAGCATC-3′

### 2.4. Immunohistochemistry (IHC) Staining

4 *μ*m thick FFPE NPC slides were used to perform IHC staining. Firstly, the tumor slides were deparaffinized. Antigen retrieval procedure was then performed in a pressure cooker in 1 mM sodium citrate (Sangon Biotech, Shanghai, China) for 1.5 minutes.* PSPH* rabbit polyclonal (Proteintech, Wuhan, China) was diluted with SigalStain antibody diluent (Cell Signaling Technology, Danvers, MA, USA) at 1:400 for 1 h. Universal secondary antibody (Gene Tech, Shanghai, China) was applied for 15 minutes. Subsequently, diaminobenzidine was used as chromogens and slides were counterstained with haematoxylin before mounting.* PSPH* IHC was scored according to the scoring criteria including intensity and percentage as follows: 0, no staining; 1+, faint cytoplasmic reactivity without any background staining; 2+, moderate cytoplasmic reactivity; and 3+, granular cytoplasmic reactivity of strong intensity; 0, 0-25%; 1, 25%-50%; 2, 50%-75% [17].

### 2.5. siRNA Transfection

Small interfering RNAs which targeted the fusion gene (si-SP1 5′-GUUGUUTTTTCCTCCCUGG-3′; si-SP2 5′-UUGUUTTTTCCTCCCUGGC-3′) were bought from RIBOBIO Company (Guangzhou, China). CNE1 and 6-10B cells were transfected with oligonucleotides (20 nmol/L) using the Lipofectamine RNAiMAX transfection reagent (Invitrogen, Carlsbad, CA, USA) according to the manufacturer's instructions.

### 2.6. Western Blotting

Cell lysis was carried out in RIPA buffer including protease inhibitor and phosphatase inhibitor cocktails (Biostool, Shanghai, China). The protein extracts were isolated from the same amounts using 12% SDS-PAGE gels. After that all the abstracts were transferred to polyvinylidene fluoride membranes (Merck Millipore, Billerica, MA, USA). Subsequently, the membranes were incubated with rabbit polyclonal anti-*PSPH* antibody 1:1000 (Proteintech, Wuhan, China). And then, the incubation was performed with goat anti-rabbit IgG antibody 1:5000 (Abcam, Cambridge, UK). Anti-*β*-actin antibody (Affinity, Ancaster, Canada) was acted as a protein control.

### 2.7. Cell Proliferation Assay

Cell proliferation was evaluated by conducting CCK-8 assays. 1000 cells were seeded per well in 96-well plates in triplicate. After incubation with 10uL of CCK8 agent (Dojindo, Kumamoto, Japan) for 3 hours in the dark in an incubator at 37°C, the absorbance value was measured at 450 nm every 12 hours.

### 2.8. Boyden Chamber Assays

Invasion and migration abilities were examined after the siRNA of* SEPT7P2-PSPH* transfection. Boyden chambers (Corning, New York, USA) with 8 *μ*m inserts equipped with (invasion) or without (migration) Matrigel were placed in the 24-well plate. A total of 5 × 10^4^ cells were suspended in 200 *μ*L RPMI 1640 without serum in the upper chambers and cultured at 37°C for 24 hours. The cells which went through the inserts were stained with 0.5% crystal (Sangon, Shanghai, China). Then, the number of cells per field of view was counted under phase-contrast microscopy.

### 2.9. Colony Formation Assay

500 transfected cells were plated in six-well plates and cultured for 7 or 12 days. All the colonies were fixed with 4% paraformaldehyde and stained with 0.5% crystal violet (Sangon, Shanghai, China) for quantification. All the experiments were performed in triplicate.

### 2.10. Anchorage-Independent Assay

In the 6-well plate, the bottom layers were the mixture of equivalent volumes of 1.2% agarose (Sangon, Shanghai, China) in PBS and medium containing 20% FBS. 5000 cells were suspended in the upper layer containing 1 ml 0.4% soft agarose.

### 2.11. Statistical Analysis

All statistical analyses were performed using SPSS 16.0 software (IBM, Armonk, NY, USA) and GraphPad Prism version 5.0 (GraphPad Software). The data are presented as the mean ± SEM from at least three independent experiments. Two-tailed Student's* t*-tests were conducted to compare the difference between groups. Progression-free survival (PFS) and overall survival (OS) were calculated using the Kaplan-Meier method, and the survival curves were compared with the log-rank test method. All tests were two-sided, and P < 0.05 was considered as statistically significant.

## 3. Results

### 3.1. Novel Fusion Genes Discovered in NPC

**Figure 1:**
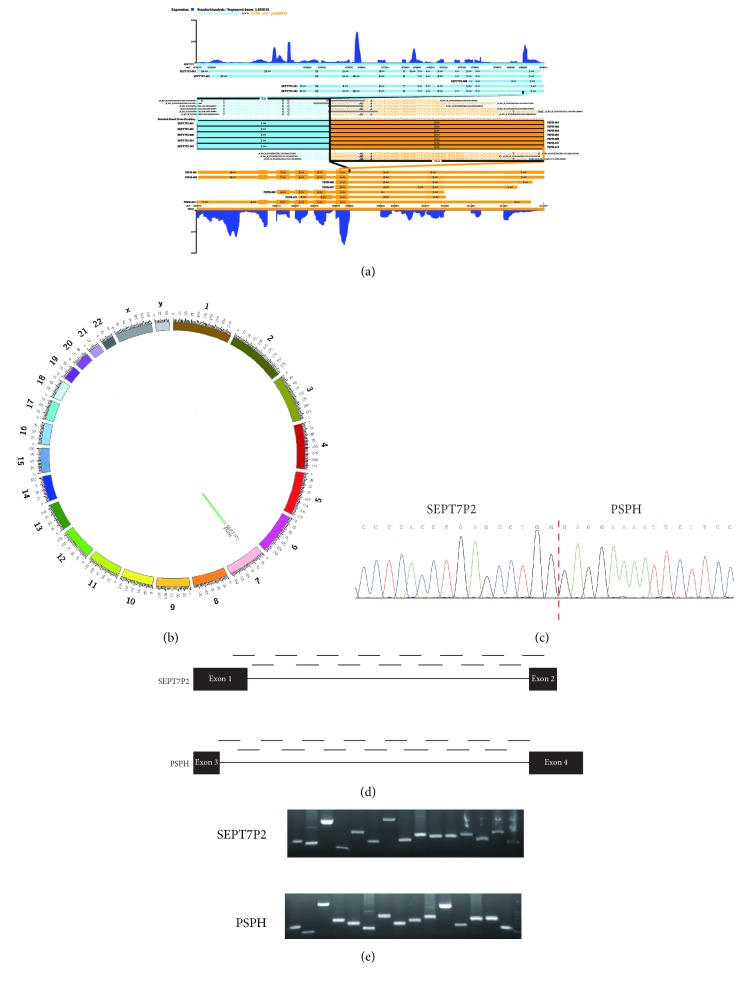
*RNA sequencing was used to discover SEPT7P2-PSPH fusion gene in NPC.* (a) Detection of* SEPT7P2-PSPH* fusion gene* via* RNA sequencing. The reads are aligned across the junction of the predicted fusion transcripts. (b) Circos plot of* SEPT7P2-PSPH* fusion gene revealed the fusion existing in chromosome 7. (c) The fusion breakpoint was verified using Sanger sequencing. (d) A schematic illustration of the amplified regions of fusion gene with exons 1-2 of* SEPT7P2* and exons 3-4 of* PSPH*. (e) The sequences between exons 1 and 2 of* SEPT7P2* and the sequences between exons 3 and 4 of* PSPH* were all amplified by RT–PCR.

We found 60 transcripts of fusion genes using RNA sequencing including 28 inter- and 32 intrachromosomal fusions from ten FFPE NPC tissues (Table 1). Paired-end reads featured by two uncorrelated genes or spanned exon-exon junctions were nominated as putative chimeric genes (Figures 1(a) and 1(b)). Fusion genes recurrently occurred in more than one sample were selected for subsequent investigation. A novel fusion gene named* SEPT7P2-PSPH* was discovered in 5 out of 10 samples and located on chromosome 7 (Figure 1(b)). To validate this newly found transcript, we designed specific primers matching the region that contained the fusion point at the mRNA level. The mRNA level of this fusion gene was validated using Sanger sequencing in the 5 samples previously and consistent with that identified by RNA sequencing (Figure 1(c)).

The transcript was formed with the exon 1 of* SEPT7P2* and the exon 4 of* PSPH*. The practical distance between the two exons are 10.2 Mb on chromosome 7. To estimate if there existed a genomic DNA rearrangement with the chimeric RNA, we designed 30 primer pairs to amplify the entire genome sequences between exon 1 and exon 2 of* SEPT7P2* as well as the genome sequences between exon 3 and exon 4 of* PSPH *(Supplementary Table 1 and Figure 1(d)). However, all the segments were amplified by PCR indirectly indicating the fact that chromosomal arrangement was absent (Figure 1(e)).

### 3.2. The Expression of the Fusion Genes in Cell Lines and Tissues

**Figure 2:**
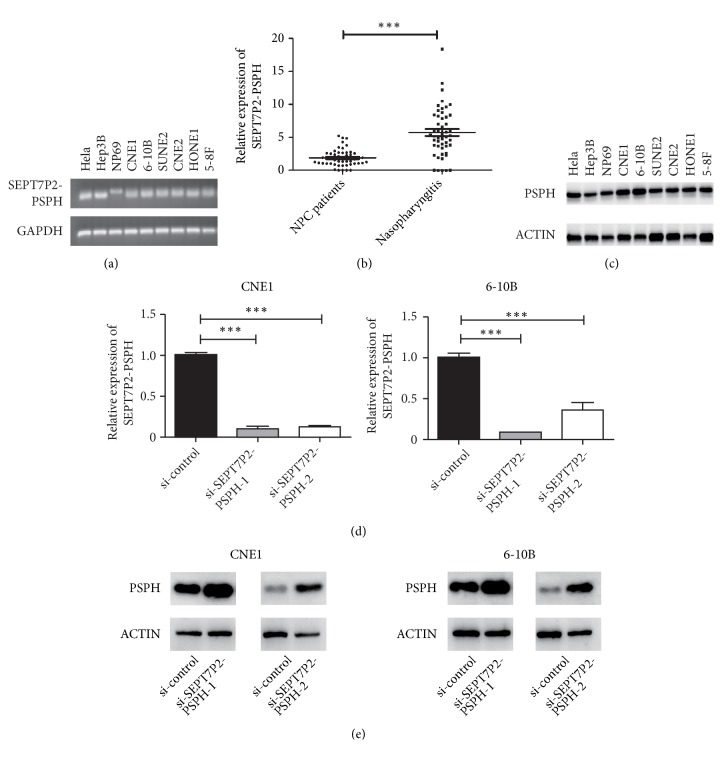
*SEPT7P2-PSPH expression*. (a) By RT–PCR, the* SEPT7P2-PSPH* fusion transcripts were verified in nine cell lines. (b) The relative expression of* SEPT7P2-PSPH* was higher in nasopharyngitis compared with those in NPC patients using quantitative RT-PCR (P < 0.05). (c) Full-length* PSPH* protein was expressed in nine cell lines by western blotting and no relevant chimeric fusion protein was detected. Expression of* SEPT7P2-PSPH* was knocked down by the specific siRNA. The suppression effect was verified by quantitative RT-PCR and western blotting, respectively (d, e, n = 3; two-tailed Student's t-tests, ^*∗∗*^*P* < 0.01 and ^*∗∗∗*^*P* < 0.001).

In addition, we have investigated several tumor cell lines including Hela, Hep3B, NP69, CNE1, 6-10B, SUNE2, CNE2, HONE1, and 5-8F. All the cell lines were confirmed to harbor the fusion gene (Figure 2(a)). Interestingly, we found that the relative expression of* SEPT7P2-PSPH* was higher in the cohort of nasopharyngitis than that in NPC patients (Figure 2(b), P < 0.05). Collectively, these results indicated that the newly found transcript was not peculiar to tumor cells. Analogous studies have been published to demonstrate the noncanonical chimeric transcript [10–12]. Simultaneously, we performed western blotting assay to discover if there would be a new type of fusion protein accompanied by the transcript. Anti-*PSPH* polyclonal antibody was applied in the nine tumor cell lines. It was shown that the putative fusion protein did not exist and only the protein of full-length* PSPH* was confirmed by western blot with the band at 25 kDa (Figure 2(c)).

### 3.3. Knocking Down* SEPT7P2–PSPH* Upregulated the Level of* PSPH* Protein and Promoted Cell Proliferation and Migration

We carried out* in vitro* experiments to confirm the potential biologic function of the* SEPT7P2-PSPH* transcript. NPC cell lines CNE1 and 6-10B were selected to explore the effect of* SEPT7P2-PSPH* on NPC tumorigenesis. When compared with the negative control group, the expression of the chimeric transcript substantially decreased after two specifically designed siRNAs, siSP1 and siSP2, were transiently transfected into the cell lines. Also, there was no novel fusion protein synthesized. After transfection for 48 hours, the expression of* SEPT7P2-PSPH* at mRNA level was decreased sharply whereas the expression of* PSPH* protein was upregulated in both cell lines (Figures 2(d)-2(e) and Supplementary Figure 1). This may indirectly show the phenomenon that the chimeric RNA fusion did not produce a new fusion protein.

**Figure 3:**
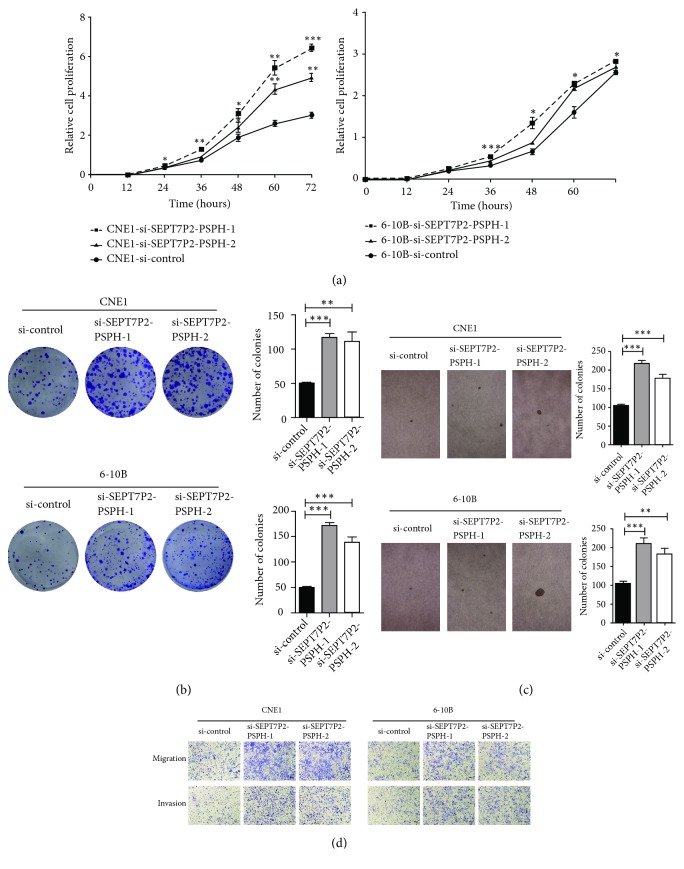
*Knocking down SEPT7P2-PSPH can promote cell proliferation and colony formation of NPC cells.* (a) CCK8 assays showed that cell proliferation was significantly enhanced after the specific siRNA interfered with the expression of* SEPT7P2-PSPH* (n = 3; two-tailed Student's t-tests, ^*∗*^*P* < 0.05, ^*∗∗*^*P* < 0.01 and ^*∗∗∗*^*P* < 0.001). (b) and (c) Knocking down* SEPT7P2-PSPH *by siRNA can promote the colony-formation ability of CNE1 and 6-10B cells (n = 3; two-tailed Student's t-tests, ^*∗∗*^*P* < 0.01 and ^*∗∗∗*^*P* < 0.001). (d) Cell migration and invasion assays revealed that the abilities of migration and invasion were enhanced after interfering with specific siRNA.

However, the expression level of down-stream gene's protein was reversely influenced by the occurrence of the fusion between* SEPT7P2 *and* PSPH*. We subsequently performed cell proliferation assays to explore the effects of the fusion gene on the growth of CNE-1 and 6-10B. The cells transfected with si-*SEPT7P2–PSPH-*1 (siSP1) and si-*SEPT7P2–PSPH-*2 (siSP1) grew faster than those with negative control (Figure 3(a), P < 0.05). Low expression of* SEPT7P2-PSPH* may contribute to tumor cell proliferation. For investigating its influence on the transforming ability of NPC cells, we conducted both colony formation assay and anchorage-independent growth assay. Strikingly, CNE1 and 6-10B cells with siSP1 and siSP2 formed larger colonies than the negative control groups, which was reversely correlated with the expression of the chimeric RNA (Figures 3(b) and 3(c), P < 0.05). To evaluate its biological function with migration and invasion, Boyden chamber assays* in vitro* have been conducted. As shown in Figure 3(d), knocking down* SEPT7P2-PSPH* with siSP1 and siSP2 markedly enhanced the migration and invasion of NPC cells (P < 0.05).

### 3.4. *PSPH* Expression and Prognosis of NPC Patients

**Figure 4:**
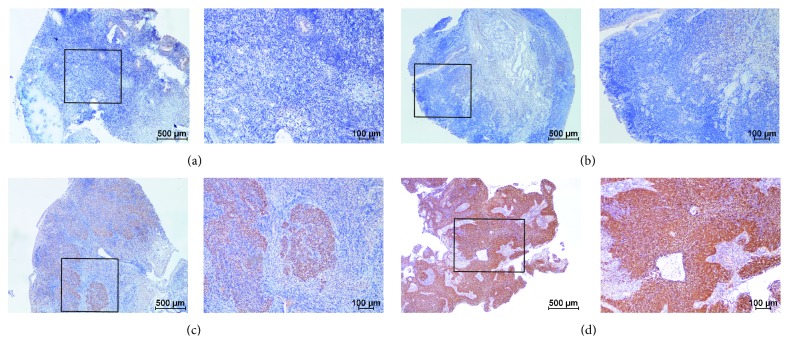
Representative IHC staining with negative (a), low (b), moderate (c), high, and (d)* PSPH* expression. Scale bar, left panel 500 *μ*m; right panel 100 *μ*m.

**Figure 5:**
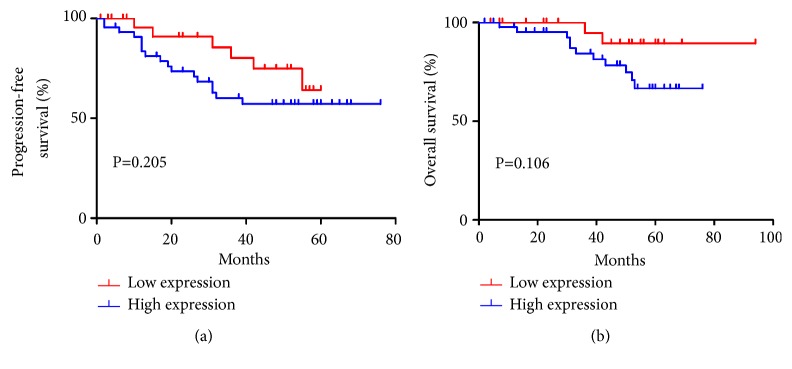
*Survival analysis.* (a) and (b) Kaplan-Meier analysis of progression-free survival and overall survival for NPC patients with low (n=27) versus high (n=45) expression of* PSPH* through immunohistochemistry (IHC) staining.* P* value was determined by the log-rank test (P > 0.05).

To investigate the expression status of* PSPH* in NPC, we performed IHC experiment for* PSPH* in 72 FFPE NPC specimens (Figure 4). The result revealed that the patients with high PSPH expression showed a tendency with shorter PFS and OS in NPC patients though there was no statistically significant (Figures 5(a) and 5(b), P = 0.205, P = 0.106).

## 4. Discussion

The present study is the first to identify trans-splicing chimeric transcript* SEPT7P2-PSPH* without chromosomal rearrangement in NPC patients. Knocking down of this transcript may promote cell proliferation and metastasis/invasion possibly due to the upregulation expression of the downstream gene* PSPH*.

Traditionally, chromosome rearrangements can generate fusion genes which are in an intimate relationship with tumor carcinogenesis. To date it makes possible to discover more and more valuable fusion genes acting as a crucial regulator in cancer formation, maintenance, and evolution. Previous studies have shown that chimeric RNAs in normal and cancer cells can be generated by intergenic splicing in the absence of chromosome rearrangements [7–9, 18, 19]. This kind of fusion gene can be used as “noncanonical chimeras”. It has been reported that there are two methods for “noncanonical chimeras” formation involving cis-splicing and trans-splicing of adjacent genes [10]. Cis-splicing fusions tend to occur between two genes that are located within 30kb in the same chromosome. Trans-splicing can be classified into two types: intragenic trans-splicing taking place between two pre-mRNAs transcribed from the same genome loci and intergenic trans-splicing that is from two different genome loci.* SEPT7P2-PSPH* can be classified as intergenic trans-splicing located on chromosome 7 aligned with the fact that the up- and downstream genes are 10.2Mb distance.

Furthermore, we found that metastasis/invasion and proliferation of the tumor cells were inversely enhanced after the transcript had been knocked down. This was different from previously analogous studies [14, 20]. The western blot result showed that there was no newly synthetic fusion protein accompanied with the chimeric RNA. Only the* PSPH* protein was translated from the downstream gene* PSPH* and was overexpressed after the chimeric RNA had been knocked down. The sequence of the chimeric RNA indicated that both of the two genes' start codons were retained after the occurrence of the fusion, one in exon 1 of* SEPT7P2* and another in exon 4 of* PSPH*. It seemed that the start codon in exon 1 of* SEPT7P2* depressed the transcription of* PSPH*. The reason why fusion genes have the ability of oncogenesis can be illuminated that the new synthesized fusion products possess the tumorigenic function. Another reason is that the preoncogene could be upregulated after connected to a stronger promoter or the antioncogene could be downregulated after fused with a weaker promoter [21].

*SEPT7P2* is a pseudogene which has been proved to be related to SEPT7 and expressed in all tissue types [22], playing a role in multiple biological processes including vesicle trafficking, apoptosis, remodeling of the cytoskeleton, neurodegeneration, and neoplasia.

Some researchers have reported that abnormal expression of* PSPH* is strongly relevant to the hepatocellular carcinoma patients' mortality as well as the critical importance on* cMyc*-induced cancer progression both* in vitro *and* in vivo *[23]. The level of* PSPH* has the relationship with breast cancer and lacrimal gland adenoid cystic carcinoma [24, 25]. In our study, high expression of* PSPH* was the tendency to NPC patients' poor survival though there was no statistically significant. Two previous studies indicated that* PSPH* was abundantly expressed in proliferating embryonic and hematopoietic stem cells and neural progenitors of the developing brain [26, 27]. Furthermore, inhibition of* PSPH* induced programmed cell death in tonsillar cell cultures [28]. In another words,* PSPH* plays an important oncogenic role in regulating the ability of cell proliferation and apoptosis. Hence, we put forward the hypothesis that it could be the* PSPH* overexpression induced when the pseudogene* SEPT7P2* and* PSPH* fuse with each other. Recently, some studies demonstrated that trans-splicing can become one of the tumor-targeting methods in cancer related treatment, in which trans-splicing ribozyme may function as a potential anticancer agent via stimulating anticancer gene activity [29]. However, such role of the transcript* SEPT7P2*-*PSPH* in NPC warrants the further study.

## 5. Conclusions

The trans-splicing chimeric transcript* SEPT7P2*-*PSPH* in our study might be a tumor suppressor gene in NPC tumorigenesis, potentially having the role of anticancer activity.

## Figures and Tables

**Table 1:** 60 transcripts of fusion genes discovered by NGS including 28 inter- and 32 intrachromosomal fusions from ten FFPE NPC tissues.

up_gene	up_chr	down_gene	down_chr	Fusion_Type
IGKC	chr2	CALR	chr19	INTERCHR-DS
IGKJ5	chr2	CALR	chr19	INTERCHR-DS
MALAT1	chr11	RFWD2	chr1	INTERCHR-DS
MRPS18A	chr6	C5orf25	chr5	INTERCHR-DS
RN7SL1	chr14	IGKC	chr2	INTERCHR-DS
RN7SL1	chr14	IGKJ5	chr2	INTERCHR-DS
RNU6-1	chr15	SNORD3A	chr17	INTERCHR-DS
RNU6-33	chr4	SNORD3A	chr17	INTERCHR-DS
RNU6-42	chr3	RPS3	chr11	INTERCHR-DS
RNU6-42	chr3	SNORD15A	chr11	INTERCHR-DS
RNU6-42	chr3	SNORD3A	chr17	INTERCHR-DS
SAMD12	chr8	ATP10D	chr4	INTERCHR-DS
ATP1B2	chr17	HSD17B12	chr11	INTERCHR-SS
E2F4	chr16	RPL14	chr3	INTERCHR-SS
EFCAB4A	chr11	RN7SK	chr6	INTERCHR-SS
IGHG1	chr14	NINJ1	chr9	INTERCHR-SS
IGKC	chr2	CSNK2A2	chr16	INTERCHR-SS
IGKJ5	chr2	CSNK2A2	chr16	INTERCHR-SS
MIDN	chr19	ALMS1	chr2	INTERCHR-SS
PDE4B	chr1	PPP6R3	chr11	INTERCHR-SS
POU2AF1	chr11	PARP11	chr12	INTERCHR-SS
RNU6-2	chr10	SNORD3A	chr17	INTERCHR-SS
RNU6-36	chr12	SNORD3A	chr17	INTERCHR-SS
RNU6-42	chr3	SNHG12	chr1	INTERCHR-SS
RPL14	chr3	GLS	chr2	INTERCHR-SS
SLC7A5P2	chr16	PHC3	chr3	INTERCHR-SS
SMARCA2	chr9	RPL14	chr3	INTERCHR-SS
ZNF827	chr4	ZNF318	chr6	INTERCHR-SS
ADCK4	chr19	NUMBL	chr19	INTRACHR-SS-OGO-0GAP
AP5S1	chr20	MAVS	chr20	INTRACHR-SS-OGO-0GAP
C12orf74	chr12	PLEKHG7	chr12	INTRACHR-SS-OGO-0GAP
COL7A1	chr3	UCN2	chr3	INTRACHR-SS-OGO-0GAP
CTBS	chr1	GNG5	chr1	INTRACHR-SS-OGO-0GAP
CTSD	chr11	IFITM10	chr11	INTRACHR-SS-OGO-0GAP
EEF1D	chr8	NAPRT1	chr8	INTRACHR-SS-OGO-0GAP
JAK3	chr19	INSL3	chr19	INTRACHR-SS-OGO-0GAP
KIAA0101	chr15	CSNK1G1	chr15	INTRACHR-SS-OGO-0GAP
LSM10	chr1	STK40	chr1	INTRACHR-SS-OGO-0GAP
MAPK7	chr17	RNF112	chr17	INTRACHR-SS-OGO-0GAP
MRPS31P2	chr13	TPTE2	chr13	INTRACHR-SS-OGO-0GAP
NPL	chr1	DHX9	chr1	INTRACHR-SS-OGO-0GAP
PIK3R2	chr19	IFI30	chr19	INTRACHR-SS-OGO-0GAP
POLA2	chr11	CDC42EP2	chr11	INTRACHR-SS-OGO-0GAP
PPCS	chr1	CCDC30	chr1	INTRACHR-SS-OGO-0GAP
PROM2	chr2	KCNIP3	chr2	INTRACHR-SS-OGO-0GAP
PSTPIP2	chr18	EPG5	chr18	INTRACHR-SS-OGO-0GAP
RRM2	chr2	C2orf48	chr2	INTRACHR-SS-OGO-0GAP
STYXL1	chr7	TMEM120A	chr7	INTRACHR-SS-OGO-0GAP
TTTY15	chrY	USP9Y	chrY	INTRACHR-SS-OGO-0GAP
VMAC	chr19	CAPS	chr19	INTRACHR-SS-OGO-0GAP
WDFY1	chr2	AP1S3	chr2	INTRACHR-SS-OGO-0GAP
WNT10B	chr12	ARF3	chr12	INTRACHR-SS-OGO-0GAP
EEF1DP3	chr13	FRY	chr13	INTRACHR-SS-OGO-1GAP
HACL1	chr3	COLQ	chr3	INTRACHR-SS-OGO-1GAP
IRF6	chr1	C1orf74	chr1	INTRACHR-SS-OGO-1GAP
TMSB4Y	chrY	KALP	chrY	INTRACHR-SS-OGO-1GAP
RMPP	chr9	SMU1	chr9	INTRACHR-SS-OGO-53GAP
FGFR3	chr4	TACC3	chr4	INTRACHR-SS-RGO
SEPT7P2	chr7	PSPH	chr7	INTRACHR-SS-RGO
SRGAP2B	chr1	SRGAP2C	chr1	INTRACHR-SS-RGO

chr, chromosome.

## Data Availability

The raw data in this paper has been successfully uploaded and locked onto Research Data Deposit with RDD no. RDDB2019000527.

## Supplementary Materials

Supplementary Materials Supplementary Figure 1. The* PSPH* expression was verified by western blotting after interfering expression of* SEPT7P2-PSPH* by the two specific siRNA. Supplementary Table 1. Primer sequences.
