# Supplementary material for: Interfering Expression of Chimeric Transcript SEPT7P2-PSPH Promotes Cell Proliferation in Patients with Nasopharyngeal Carcinoma
Source: J Oncol. 2019 Apr 1;2019:1654724. doi: 10.1155/2019/1654724 (PMC6463592; doi:10.1155/2019/1654724)
Supplement: Supplementary Materials — Supplementary Figure 1. The PSPH expression was verified by western blotting after interfering expression of SEPT7P2-PSPH by the two specific siRNA. Supplementary Table 1. Primer sequences. [file 1654724.f1.docx]

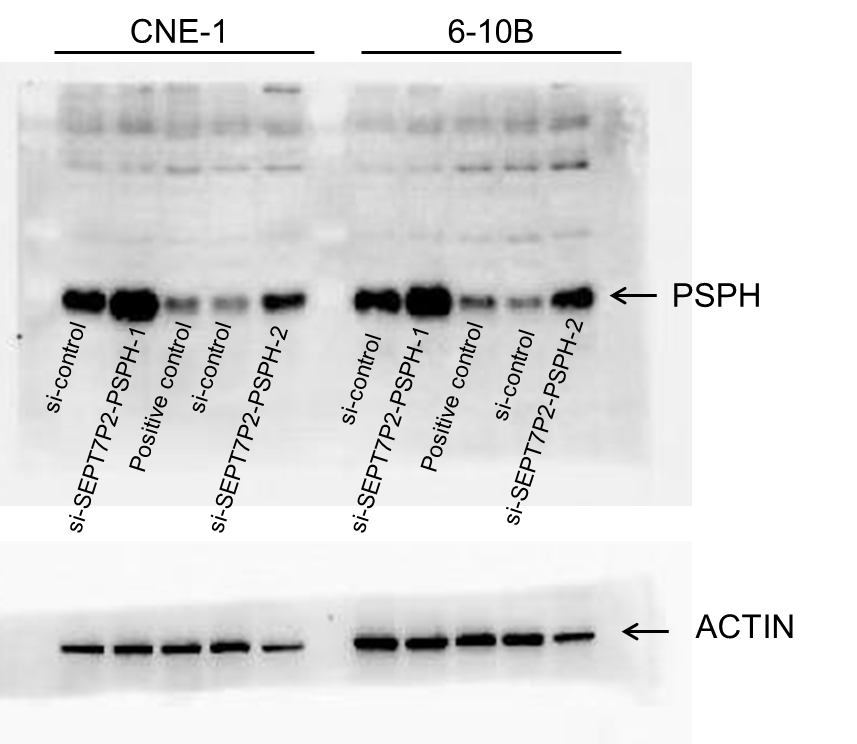


**Supplementary Figure 1**. The *PSPH* expression was verified by western blotting after interfering expression of *SEPT7P2-PSPH* by the two specific siRNA.

| **Supplementary Table1**. Primer sequences | |
| --- | --- |
| Primers ID | Sequence |
| SEPT7P2-D-1-F | AACTGCGAGGAGGAGCTTTA |
| SEPT7P2-D-1-R | TTTCCTAGCCCCTTCTACCC |
| SEPT7P2-D-2-F | AGAGCTTTTCTTCCAGCCCTC |
| SEPT7P2-D-2-R | TTGTTTGCACCACCTCACTCCA |
| SEPT7P2-D-3-F | AGATTACAGGAATGAGCCATC |
| SEPT7P2-D-3-R | TACCGCTTCTACTCAAGGTA |
| SEPT7P2-D-4-F | CCTATTATGCTAAGTGAGT |
| SEPT7P2-D-4-R | AATCTGCTAATACAAAGAA |
| SEPT7P2-D-5-F | TTTGTGGAGAAGCACCCT |
| SEPT7P2-D-5-R | GTGGCAAATGACTATCATA |
| SEPT7P2-D-6-F | TCCTAGAGATGACACTGTGA |
| SEPT7P2-D-6-R | CATGCTCAGTCTTAGAAA |
| SEPT7P2-D-7-F | TATTAATCTTTGAAATGTGG |
| SEPT7P2-D-7-R | ATGTCACATCTTTAACAGA |
| SEPT7P2-D-8-F | CTGATTTATAGGAGTTTGAA |
| SEPT7P2-D-8-R | AATCTATTCTAGGAAACTG |
| SEPT7P2-D-9-F | GAAATAGTGTAAACGGTT |
| SEPT7P2-D-9-R | TTGCTCAAGAGGTAGCATTA |
| SEPT7P2-D-10-F | TTTCTTGTGGAACACGATGA |
| SEPT7P2-D-10-R | TAGTGCCATTCACAGGAT |
| SEPT7P2-D-11-F | CATGAGGATACTTGTAA |
| SEPT7P2-D-11-R | TTCAAACTCCTGGGTTCAAG |
| SEPT7P2-D-12-F | GACTAGAAGATCACCAGAT |
| SEPT7P2-D-12-R | AGCAGAGGTCTATAGCAA |
| SEPT7P2-D-13-F | ATCAGGATCAAAGCAGT |
| SEPT7P2-D-13-R | TTCATAAGATAGGCTT |
| SEPT7P2-D-14-F | CATGTTAGTCTCTTCATGCA |
| SEPT7P2-D-14-R | TTCAAATGTAATTAATCAG |
| SEPT7P2-D-15-F | ACTATAAACAGTAGATTA |
| SEPT7P2-D-15-R | TTACTTGAAGTTTGGTGA |
| PSPH-D-1-F | TCACACCTCCGCTTTAATGCTTG |
| PSPH-D-1-R | CGTCCTCAACGCCACAGATT |
| PSPH-D-2-F | CACATGCCAAATCGATGCCC |
| PSPH-D-2-R | GGAAGCAAGCATTAAAGCGGA |
| PSPH-D-3-F | TCTGTAGCAGTGTGGCTTCC |
| PSPH-D-3-R | GATTTGGCATGTGCTGGGAG |
| PSPH-D-4-F | AGTTTGCTGACCCCTGACTT |
| PSPH-D-4-R | GGAAGCCACACTGCTACAGA |
| PSPH-D-5-F | TGGGTACTGTGGCTCTGGTA |
| PSPH-D-5-R | TGCAGTTGGCAGAATAAGGC |
| PSPH-D-6-F | GGTGATTCCAGGCAGAGGAG |
| PSPH-D-6-R | CAGAGCCACAGTACCCAGC |
| PSPH-D-7-F | GCTGGACCAAAGCCAAATGA |
| PSPH-D-7-R | GCTCCTCTGCCTGGAATCAC |
| PSPH-D-8-F | TTGGGTCTTGTGTCAGGGTC |
| PSPH-D-8-R | TTTGGCTTTGGTCCAGCAGA |
| PSPH-D-9-F | AGGAGGCAGGTGTCAACAAT |
| PSPH-D-9-R | GAATCTCAAAGCCAGTCCCCA |
| PSPH-D-10-F | CCAATCAAGCAGGGAGGGAG |
| PSPH-D-10-R | TCCTGGGTTGTAATGTGGTCC |
| PSPH-D-11-F | GAATAGACCTTCCCACCGGC |
| PSPH-D-11-R | CTCCCTCCCTGCTTGATTGG |
| PSPH-D-12-F | GTGTAAGTGCATGTGCTGGG |
| PSPH-D-12-R | GCCGGTGGGAAGGTCTATTC |
| PSPH-D-13-F | GCAGTTTACTCCTCACCTGAA |
| PSPH-D-13-R | CCAGCACATGCACTTACACTAT |
| PSPH-D-14-F | GAACTGTTACACGTTTGTCTATCTG |
| PSPH-D-14-R | TCTTCTGCATGCCACATAGTTGA |
| PSPH-D-15-F | CTCTTGCAGAATCCACAGGTC |
| PSPH-D-15-R | ATTCTGGGAAGCTGAGATCTTCT |
